# Detection of Japanese Encephalitis Virus in *Culex* Mosquitoes in Singapore

**DOI:** 10.4269/ajtmh.19-0377

**Published:** 2020-07-20

**Authors:** Grace Yap, Diyar Mailepessov, Xiao Fang Lim, Sharon Chan, Choon Beng How, Mahathir Humaidi, Gladys Yeo, Chee Seng Chong, Sai Gek Lam-Phua, Ruth Lee, Chiharu Okumura, Indra Vythilingam, Lee Ching Ng

**Affiliations:** 1 Environmental Health Institute, National Environment Agency, Singapore;; 2 National Parks Board, Singapore;; 3 Wildlife Reserves Singapore, Singapore;; 4 Parasitology Department, Faculty of Medicine, University of Malaya, Kuala-Lumpur, Malaysia

## Abstract

Mosquito-borne flaviviruses are emerging pathogens of an increasing global public health concern because of their rapid increase in geographical range and the impact of climate change. Japanese encephalitis virus (JEV) and West Nile virus (WNV) are of concern because of the risk of reemergence and introduction by migratory birds. In Singapore, human WNV infection has never been reported and human JEV infection is rare. Four sentinel vector surveillance sites were established in Singapore to understand the potential risk posed by these viruses. Surveillance was carried out from August 2011 to December 2012 at Pulau Ubin, from March 2011 to March 2013 at an Avian Sanctuary (AS), from December 2010 from October 2012 at Murai Farmway, and from December 2010 to December 2013 at a nature reserve. The present study revealed active JEV transmission in Singapore through the detection of JEV genotype II in *Culex tritaeniorhynchus* collected from an Avian Sanctuary. *Culex* flavivirus (CxFV), similar to the Quang Binh virus isolated from *Cx. tritaeniorhynchus* in Vietnam and CxFV-LSFlaviV-A20-09 virus isolated in China, was also detected in *Culex* spp. (*vishnui* subgroup)*.* No WNV was detected. This study demonstrates the important role that surveillance plays in public health and strongly suggests the circulation of JEV among wildlife in Singapore, despite the absence of reported human cases. A One Health approach involving surveillance, the collaboration between public health and wildlife managers, and control of mosquito populations remains the key measures in risk mitigation of JEV transmission in the enzootic cycle between birds and mosquitoes.

## INTRODUCTION

Mosquito-borne flaviviruses are emerging pathogens of an increasing public health concern because of their rapid increase in incidence and geographical range.^1^ Japanese encephalitis virus (JEV), which has spread through much of Asia, and West Nile virus (WNV), which has emerged in North America and reemerged in Europe, are important emerging viruses.^1–4^ Japanese encephalitis virus and WNV are members of the JEV serocomplex of the genus *Flavivirus* of the family *Flaviviridae*. *Culex* spp. mosquitoes serve as the main vector of both viruses, whereas ardeid birds are the main reservoir host for virus maintenance and humans are accidental dead-end hosts.^1,2^

Japanese encephalitis virus is estimated to cause close to 68,000 cases annually.^5^ As pigs are important amplifying hosts for JEV, large outbreaks are known to occur in areas with intensive pig farming.^6,7^ Five genotypes of JEV are presently recognized,^8^ and all five have spread to varying degrees across South Asia, Southeast Asia, East Asia, Australasia, and the Pacific regions.^2,3^ A localized outbreak of JEV was also reported in Angola, Africa in 2016.^9^

West Nile virus, similar to JEV, may cause encephalitis in humans in severe cases.^10^ West Nile virus cases vary greatly from year to year; however, in the United States alone between 1999 and 2013, WNV caused close to 16,000 confirmed cases with more than 1,500 deaths.^11^ Unlike JEV, WNV and its subtypes have been reported from a vast region of the globe.^12^ Migratory birds have been implicated in virus introductions into new territories.^3,13–16^ The emergence and risk of these mosquito-borne diseases are reported to be linked to climatic parameters (e.g., temperature, relative humidity, and rainfall) and environmental drivers (e.g., host and vector abundances, and land use).^17,18^

Singapore, a highly urbanized island state, retains a lot of its biodiversity through conservation efforts. Hence, 56% of the island is covered with greenery that includes nature reserves (NRs), urban parks, mangroves, and adventitious vegetation.^19^ These greener areas support close to 400 species of birds,^20^ including migratory birds, and produce a conducive environment for more than 140 species of mosquitoes.^21^ Among the birds, several species of family *Ardeidae* such as black-crowned night heron, cattle egret, and little egret are known to be reservoirs for JEV.^22^ Among mosquitoes, there are several vectors of JEV and WNV, such as *Culex tritaeniorhynchus*,^2^ *Culex vishnui*,^23^ *Culex quinquefasciatus*,^24^ *Culex gelidus*,^25^ and other potential vectors of the same genus.^21^

There has been no report of WNV in humans or animals in Singapore. However, the island state resides within the JEV-endemic region in Southeast Asia and had an annual average of 14 JEV cases from 1978 to 1982.^26^ Japanese encephalitis virus incidence in humans reduced following the abolishment of pig farms in the early 1990s, with only six reported indigenous cases from 1991 to 2005.^27^ There have been no reports of locally acquired cases since 2005. Studies in the early 2000s showed the presence of JEV antibodies in wild boars, dogs, chickens, and goats in the rural regions of Singapore.^26–28^ The presence of JEV antibodies in local wild birds and wild boar populations has persisted as revealed by a more recent study.^22^ Seroconversion of sentinel chickens further supported enzootic transmission of JEV in Singapore.^22^ The detection of JEV antibodies among migratory birds in the recent study and the presence of resident host birds such as egrets and herons suggest the potential role of both migratory and residential birds in the transmission of JEV in Singapore.

Singapore is located along the East Asian–Australasian Flyway (EAAF) used by migratory birds during each migratory season from September to March. Birds fly from as far as the Arctic Circle, across East and Southeast Asia to Australia and New Zealand, to take refuge in Singapore, widely known as the “City in the Garden.” Among these birds, some members are known to be important viral reservoirs (e.g., ardeid birds) and have been implicated in the introduction of JEV genotype I to temperate regions such as Northern Vietnam and Eastern Asia (Japan and Korea) from Southeast Asia in the early 1990s.^3,13^ Ardeid birds were also implicated in the spread of the Kunjin virus (KUNV), subtype of WNV, from northern Australia to southern Australia.^16^ As Singapore is a stopover for the migratory birds, it houses an estimated 20,000 birds every season (D. Li, NParks, personal communication).

In view of the suspected enzootic transmission of JEV and the potential risk of entry of WNV via migratory birds, an entomological surveillance system was set up at four sentinel sites. Field-caught mosquitoes were screened for JEV, WNV, and flaviviruses using specific and generic PCRs. To fully understand the risk of transmission of these zoonotic flaviviruses, the surveillance was conducted throughout the year. The system also serves to characterize the vector mosquito populations at the four sentinel sites.

## MATERIALS AND METHODS

### Sentinel sites and surveillance.

**Figure 1.**
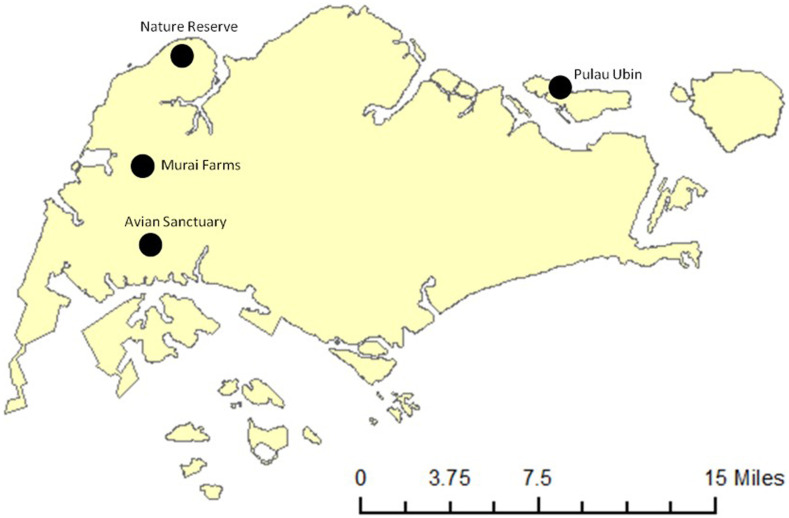
Location of the four sentinel sites on the map of Singapore. This figure appears in color at www.ajtmh.org.

Four mosquito sentinel sites were set up in 2011 at a NR, Murai Farmway (MF), Pulau Ubin (PU), and an Avian Sanctuary (AS) in the western part of Singapore (Figure 1). Sites were selected based on the relatively high abundance of animal hosts (migratory birds, resident birds, and wild boars) and mosquito vectors. The trapping was conducted throughout the year. Surveillance was carried out from August 2011 to December 2012 at PU, from March 2011 to March 2013 at an AS, from December 2010 to October 2012 at MF, and from December 2010 to December 2013 at an NR.

The nature reserve, situated in the northwestern part of Singapore (longitude: 103.730095, latitude: 1.446415), is home to numerous resident birds and is an annual stopover site along the EAAF for migratory birds during September to March. As the NR is the main site where migratory birds visit, more thorough surveillance (monthly, over three years) was carried out at this study site. Murai Farmway is in the west of the island (longitude: 103.696153, latitude: 1.392072) and is largely occupied by layer chicken farms. Pulau Ubin is an offshore island in the northeastern part of Singapore (longitude: 103.961102, latitude: 1.413264) home to a small population of humans, numerous resident birds, wild boars, and other wildlife. Pulau Ubin is also a stopover site for migratory birds. The Avian Sanctuary is a wildlife park that serves as a bird sanctuary and is also visited by migratory birds (longitude: 103.704844, latitude: 1.317557).

Mosquito trapping was performed using Centers for Disease Control light traps baited with dry ice. Each trapping session comprised two nights of 16 hours each (including crepuscular periods). Five trap locations were selected in each sentinel site. The traps were positioned approximately 1.5 m above ground in shaded areas.

### Mosquito identification.

Mosquitoes were collected after each 16-hour trap night and transported on dry ice to the Environmental Health Institute, a public health laboratory under the National Environment Agency of Singapore. Mosquitoes were sorted and identified to species where possible and to species group for morphologically similar species using relevant mosquito identification keys.^29–34^ Damaged specimens were identified to the genus level. After identification and enumeration, females from six *Culex* vector species—*Culex bitaeniorhynchus*, *Cx. gelidus*, *Cx. quinquefasciatus*, *Culex sitiens*, *Cx. tritaeniorhynchus*, and *Culex* spp. (*vishnui* subgroup)—were pooled according to species, in groups of 10–50, and kept at −80°C until further analysis.

### Virus isolation.

Pooled mosquito samples were homogenized in 500 µL of virus transport media (Copan Diagnostics, Murrieta, CA) using the Mixer Mill MM 400 (Retsch Technology GmbH, Haan, Germany). Virus transport media was used to preserve the viruses in mosquito pool lysates that were kept at −80°C. A portion of the homogenate was used for virus isolation, and the rest was kept for genome analysis. The entire homogenate was filtered (Minisart 0.45 microns, Sartorius, Göttingen, Germany) before inoculation into C6/36 cells (ATCC CRL-1660). C6/36 cells were incubated in Leibovitz L-15 media (Thermo Fisher Scientific Inc., Waltham, MA) with 2% fetal bovine serum (Thermo Fisher Scientific Inc.) at 33°C and monitored for a week before conducting immunofluorescence assay (IFA).^35,36^

### Immunofluorescence assay.

Immunofluorescence assay was performed using an anti-*Flavivirus* antibody (ATCC HB-112) to detect the presence of any flavivirus replication. Cells were scraped and washed in 200 µL PBS. Two microliters of the cell suspension were spotted onto teflon slides and left to dry. On drying, the slides were fixed in 80% acetone for 5 minutes. Anti-*Flavivirus* antibody (ATCC HB-112) together with secondary anti-mouse Ig-FITC (Bethyl Laboratories Inc., Montgomery, TX) was used to detect the presence of any flavivirus infection.

### RNA extraction and reverse transcriptase PCR (RT-PCR).

Viral RNA was extracted from the mosquito homogenate using AllPrep DNA/RNA Mini Kit (Qiagen Group, Hilden, Germany) according to the manufacturer’s instructions. One microliter of RNaseOUT, a ribonuclease inhibitor (Life Technologies Corp., Carlsbard, CA), was added to each sample after the final step of the RNA extraction. RNA was kept at −80°C until further use.

Positive RNA controls of JEV (Nakayama) and WNV (ATCC 1507, NY-99) strains were extracted from Vero cells (ATCC CCL-81) supernatant using a QIAmp Viral RNA Mini kit (Qiagen Group) according to the manufacturer’s recommendations. One hundred forty microliters of cell culture supernatant was used for each extraction, and RNA was eluted in 60 µL. For positive control reactions, RNA extracts were diluted to achieve higher cycle threshold (ct) values, reducing the possibility of cross-contamination.

A duplex real-time JEV and WNV RT-PCR protocol was optimized on a LightCycler^®^ 2.0 (Roche Life Science, Penzberg, Germany) based on the protocols by Scherret et al. and Santhosh et al.^37,38^ A pan-flavivirus PCR from Yang et al.^39^ was also performed on the mosquito pools. Primers from Yang et al., Scherret et al., and Santhosh et al. are summarized in Table 1. Pan-flavivirus primers targeted region 9007-9215 within the NS5 gene based on JEV isolate Bennett sequence (GenBank accession no. HQ223285.1). KUNV primers targeted region 1210-1706 within E gene based on WNV strain 385-99 (GenBank accession no. AY848696.2). Japanese encephalitis virus primers were originally designed to amplify 5739-5900 region within the NS3 gene based on prototype strain JaOArS982 (GenBank accession no. M18370). In brief, the reaction occurred in a final volume of 20 µL, consisting 5 µL of RNA template and a concentration of 10 µM for each primer in a Lightcycler 2.0 (Roche Diagnostics GmbH, Mannheim, Germany). The cycling conditions were as follows: reverse transcription at 50°C for 20 minutes, inactivation at 95°C for 15 minutes, and subsequently 40 cycles of 94°C for 15 seconds, 55°C (pan-flavivirus), or 58°C (Japanese encephalitis-west nile [JE-WN]) for 30 seconds and 72°C for 30 seconds. Melting curve analysis was conducted at 65°C for 15 seconds followed by a cooling step at 37°C for 20 seconds to verify the amplified products. The cutoff for positive samples was set at ct35 with melting peaks that match the controls.

**Table 1:** Primers for pan-flavivirus rRT-PCR and JE-WN duplex rRT-PCR

Primer	Sequence (5′-3′)	Tm (°C)	Amplicon size (bp)	Genomic region
Pan-flavivirus rRT-PCR (Yang et al.)				
F1	GCC ATA TGG TAC ATG TGG CTG GGA GC	80–84	209	9007-9029 (NS5)
R3	GTK ATT CTT GTG TCC CAW CCG GCT GTG TCA TC			9215-9184 (NS5)
R4	GTG ATG CGR GTG TCC CAG CCR GCK GTG TCA TC			9215-9184 (NS5)
JE-WN duplex rRT-PCR (Sherret et al. and Santhosh et al.)				
KUN5276	ATA ATG ACA AGC GGG CTG ACC C	83–84	497	1210-1229 (E gene)
KUN4778	GCG TGT GGT TCT TCA AAC TCC A			1706-1685 (E gene)
JE-F	AGA GCG GGG AAA AAG GTC AT	80–81	162	5739-5758 (NS3)
JE-R	TTT CAC GCT CTT TCT ACA GT			5900-5881 (NS3)

JEV = Japanese encephalitis virus; RT-PCR = reverse transcriptase PCR. The genomic regions for pan-flavivirus PCR are based on JEV isolate Bennett sequence (GenBank accession no. HQ223285.1). For KUNV and JEV primers, West Nile virus strain 385-99 (GenBank accession no. AY848696.2) and JaOArS982 (GenBank accession no. M18370) were used.

### Verification of duplex and pan-flavivirus RT-PCRs.

Verification of the duplex and pan-flavivirus RT-PCRs was performed using JEV (Nakayama) and WNV (ATCC 1507, NY-99) strains. Both viruses were grown in Vero cells (ATCC CCL-81) in a biosafety level-3 facility at the Environmental Health Institute. On observing the cytopathic effect at around 80%, cells were harvested and pelleted by centrifugation at 3,000 × *g* for 5 minutes. The virus supernatant was then aliquoted and stored at −80˚C for later use. The titer of the virus was measured using plaque assay as the number of plaque forming units per milliliter (PFU/ml). The titers of JEV and WNV stock viruses were 1.5 × 10^6^ and 2.5 × 10^6^ PFU/mL, respectively. RNA extraction was performed as described earlier. RNA extracts for both viruses were first diluted to 10^6^ PFU/mL and then 10-fold to achieve dilutions equivalent to 10^4^ to 1, based on original virus stock. These were then used as templates for the duplex PCR and single PCRs on their own. For each reaction, 5 µL of the RNA template was added.

### Sequencing the envelop gene (*E* gene) of JEV.

The envelope (*E*) gene of JEV-positive pools was amplified as described elsewhere.^40^ Products were purified using the QIAquick PCR purification kit (Qiagen) and sequenced at a commercial sequencing facility according to the BigDye terminator Cycle Sequencing Kit (Applied Biosystems). Raw sequence data were assembled using Lasergene package version 7 (DNAStar Inc., Madison, WI). Multiple sequence alignments and phylogenetic analyses were performed using MEGA 6.06 software suite. A phylogenetic tree was constructed using the neighbor-joining method based on the Kimura 2-parameter substitution model with gamma-distributed rates and 1,000 bootstraps reiterations.^41^ Reference sequences were obtained from GenBank. The newly generated sequences in the present study were deposited in the GenBank nucleotide database (Accession no. KR902541 to KR902545).

### Sequencing of flavivirus nonstructural 5 gene (NS5 gene).

The viral RNA was reverse transcribed by SuperScript Reverse Transcriptase (Life Technologies) according to the manufacturer’s protocol. PCR was performed using 1X Phusion™ Flash High-Fidelity PCR Master Mix (Thermo Scientific) using FU2 (5′_9233 GCTGATGACACCGCCGGCTGGGACAC 9259_3′) and CFD3 (5′_10077AGCATGTCTTCCGTGGTCATCCA10100_3′) primers that flank 844 bp at the N-terminal of the NS5 gene.^42^ The amplicons were purified by the QIAgen PCR purification kit according to the manufacturer’s protocol and subjected to sequencing at a commercial sequencing facility using the BigDye terminator Cycle Sequencing Kit (Applied Biosystems, Foster city, CA). Phylogenetic analysis of the NS5 gene was performed using the neighbor-joining method as described earlier.

## RESULTS

### Verification of duplex and pan-flavivirus RT-PCRs.

The sensitivity of duplex PCR was similar to what was observed in each single PCRs (Table 2). The estimated detection limits at ct35 for JEV were 0.4 PFU/mL for single PCR and 1 PFU/mL for duplex PCR. Similarly, at ct35 for WNV, the estimated limits of detection were recorded at 1.1 PFU/mL and 1.7 PFU/mL for single and duplex PCR, respectively.

**Table 2:** Single PCRs, Duplex PCR, and pan-flavivirus PCR sensitivity analysis

	Results obtained with			Results obtained with			
JEV standard (PFU/mL)	JEV primers only	Duplex PCR	Pan-flavivirus PCR	WNV standard (PFU/mL)	WNV primers only	Duplex PCR	Pan-flavivirus PCR
104	15.76	15.44	22.07	10^4^	17.31	20.07	24.89
103	19.64	20.10	25.65	10^3^	21.78	25.91	30.16
102	23.60	26.42	29.51	10^2^	27.09	29.17	34.29
101	29.46	29.50	> 35.00	10^1^	30.26	31.27	> 35.00
1	> 35.00	> 35.00	> 35.00	1	> 35.00	> 35.00	> 35.00

JEV = Japanese encephalitis virus; WNV = West Nile virus. The standard RNA templates are 10-fold dilutions of JEV and WNV RNA extracts of original stocks with titers ∼106 PFU/mL (only 104-1 PFU/mL were used). Detection of JEV in mosquitoes.

The sensitivity of pan-flavivirus PCR compared with duplex PCR was significantly lower (Table 2). The detection limits at ct35 were estimated to be at 2.9 PFU/mL for JEV and 77.5 PFU/mL for WNV.

**Figure 2.**
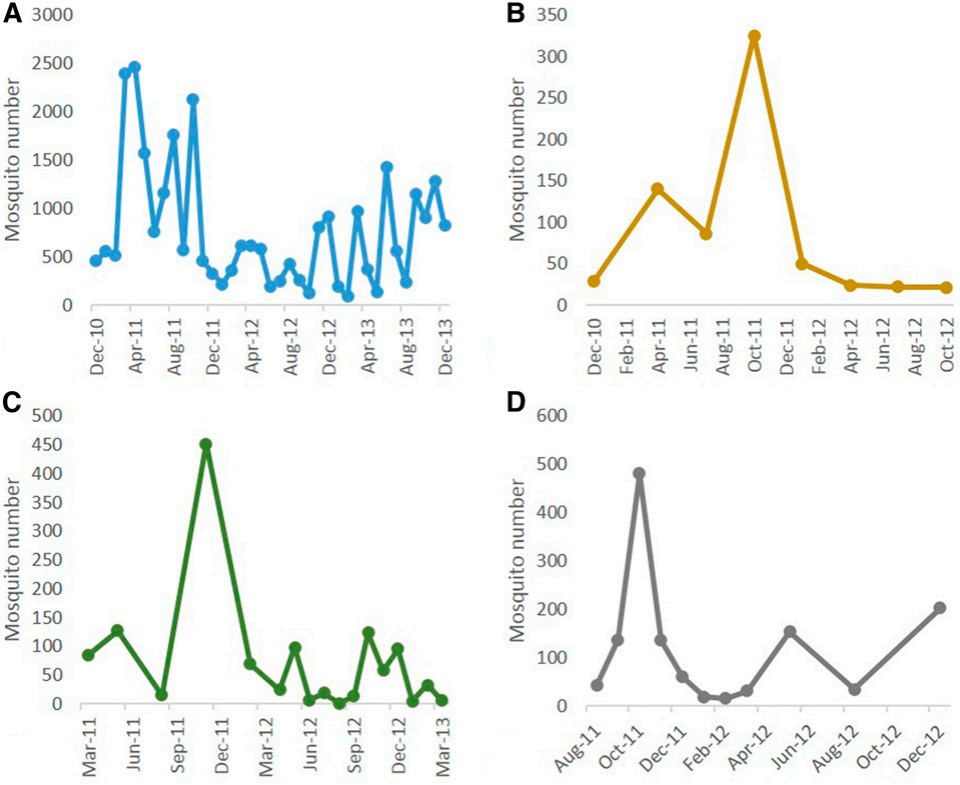
Mosquito species abundance at the four sentinel sites throughout the study period. (**A**) Nature reserve, (**B**) Murai Farmway, (**C**) Avian Sanctuary, and (**D**) Pulau Ubin. This figure appears in color at www.ajtmh.org.

A total of 31,557 mosquitoes (1,829 pools) were screened, comprising *Culex* species that are known to be or are suspected vectors of JEV and WNV: 126 *Cx. bitaeniorhynchus*, 155 *Cx. gelidus*, 281 *Cx. quinquefasciatus*, 1,698 *Cx. sitiens*, 882 *Cx. tritaeniorhynchus*, and 28,315 *Culex* spp. (*vishnui* subgroup) (Table 3). The temporal abundance of these species at each sentinel location is depicted in Figure 2. *Culex* spp. (*vishnui* subgroup) was the most common species at all four sites, comprising 90.4% of all the *Culex* spp. trapped.

**Table 3:** Number of *Culex* species mosquitoes collected at the four sentinel sites included in this study

	Nature reserve (%)	Murai Farmway (%)	Avian Sanctuary (%)	Pulau Ubin (%)	Total number
*Culex (Culex) bitaeniorhynchus*	116 (0.4)	8 (1.2)	1 (0.1)	1 (0.1)	126
*Culex (Culex) gelidus*	25 (0.1)	64 (9.3)	9 (0.5)	57 (6.9)	155
*Culex (Culex) quinquefasciatus*	307 (1.1)	4 (0.6)	9 (0.5)	61 (7.4)	381
*Culex (Culex) sitiens*	1,559 (5.5)	37 (5.4)	2 (0.2)	100 (12.2)	1,698
*Culex (Culex) tritaeniorhynchus*	176 (0.6)	82 (11.9)	614 (35.8)	10 (1.2)	882
*Culex* spp. (*vishnui* subgroup)	26,146 (92.3)	496 (71.8)	1,079 (63.0)	594 (72.2)	28,315
Total	28,329	691	1,714	823	31,557

Trapping was performed from December 2010 to December 2013 at the nature reserve, from December 2010 to October 2012 at Murai Farmway, from March 2011 to March 2013 at the Avian Sanctuary, and from August 2011 to December 2012 at Pulau Ubin.

**Figure 3.**
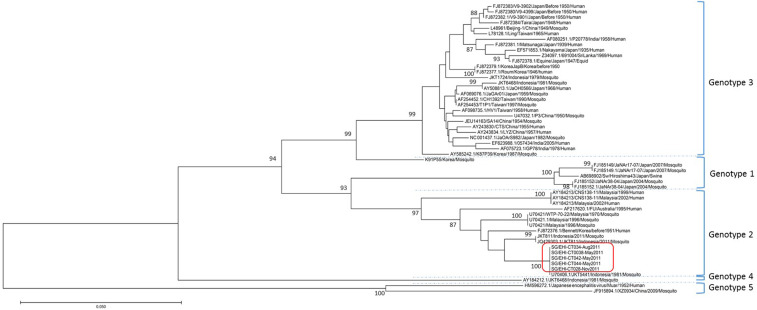
Neighbor-joining tree of Japanese encephalitis virus strains using complete *E* gene nucleotide sequence homologies. Viruses detected in *Culex tritaeniorhynchus* at the Avian Sanctuary are highlighted by the red box. The scale bar shows the number of substitutions per site. This figure appears in color at www.ajtmh.org.

Of all mosquitoes trapped, only *Cx. tritaeniorhynchus* was positive for JEV. Of the 88 pools of *Cx. tritaeniorhynchus* screened, five (5.7%) pools were JEV positive (denoted as SG/EHI-CT, -28, -34, -38, -42 and -44). All JEV positive samples were collected from the AS between May and November 2011 (Accession no. KR902541 to KR902545). E gene–based phylogeny revealed that all five positive pools contained identical E gene sequences of JEV genotype II which were closely related to those circulating in Indonesia in the same year and 1981 (Figure 3). None of the mosquitoes collected after November 2011 was JEV positive. None were WNV positive.

**Figure 4.**
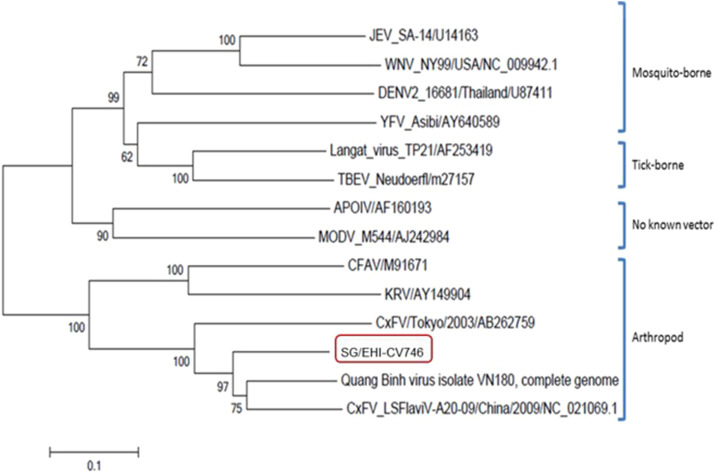
Phylogenetic analysis based on about 800 deduced nucleotides of the NS5 gene of the mosquito flavivirus detected in *Culex* spp. (*vishnui* subgroup) from the nature reserve. SG/EHI-CV746 is highlighted by the red box. The scale bar shows the number of substitutions per site. This figure appears in color at www.ajtmh.org.

A mosquito flavivirus (denoted as SG/EHI-CV746) was detected in *Culex* spp. (*vishnui* subgroup) from the NR using the pan-flavivirus PCR. An initial BLAST search (discontiguous megablast) showed that this flavivirus has the closest hit (80% identity) with a Quang Binh virus isolate from Vietnam in 2002 (Accession no. FJ644291.1). Phylogenetic analysis of the NS5 gene of this flavivirus showed that SG/EHI-CV-746 formed a sister branch with Quang Binh virus isolated from *Cx. tritaeniorhynchus* in Vietnam in 2002 and with *Culex* flavivirus (CxFV)-LSFlaviV-A20-09 virus isolated in China. *Culex* flavivirus, as its name suggests, is only found in *Culex* mosquitoes (Figure 4)*.* SG/EHI-CV746 could not be isolated following one passage through C6/36.

## DISCUSSION

The sensitivity of the duplex RT-PCR for JEV and WNV was comparable with the individual single PCRs. The sensitivity of pan-flavivirus PCR compared with duplex PCR was lower by at least one log in PFU/mL. Pan-flavivirus PCR is valuable in the screening of mosquitoes for a wide variety of flaviviruses; however, the virus must be present in relatively high titers in mosquito pools to be detected. Also, the predicted detection limit for JEV in pan-flavivirus PCR at ct35 was slightly lower than that in the actual test. This is likely because of the imperfect curve derived from the three dilutions (10^4^, 10^3^, and 10^2^ PFU/mL) and possible pipetting error.

In the absence of clinically diagnosed cases, mosquito surveillance can offer an insight into the presence of WNV and JEV in Singapore. This study represents the first detection of JEV in field-caught mosquitoes in Singapore. Together with the recent report on the detection of JEV antibodies in resident and migratory birds, wild boars, and sentinel chickens, the data suggest JEV is enzootic in Singapore.^22^ Sequencing of the envelope gene of JEV revealed the identical virus in all five pools of mosquitoes, suggesting localized transmission at the AS in 2011. As the mosquitoes were accumulated and processed within a relatively short period, there is a remote possibility of cross-contamination with positive pools. However, care was taken to minimize such instances, for example, including blank controls during extraction. Detected JEV was found to belong to genotype II which was closely related to a strain in *Cx. tritaeniorhynchus* in Jakarta, Indonesia, in 1981 and 2011. Japanese encephalitis virus genotype II strains have been isolated sporadically between 1951 and 1999 in Korea, Southern Thailand, Malaysia, Indonesia, Papua New Guinea, and northern Australia.^43^

Avian Sanctuary has a favorable environment for JEV transmission, with the presence of JEV vectors and ardeid water birds (black crown night herons, grey heron, egrets, and bitterns) which are known reservoir hosts of the virus.^3,13^ In addition, the AS registered a sizable population of *Cx. tritaeniorhynchus* which is the primary vector of JEV. As *Cx. tritaeniorhynchus* is a nocturnal biter and active at night, the absence of human hosts at the AS at night may explain the absence of human JEV cases in this area. The Avian Sanctuary is also situated away from residential neighborhoods, further minimizing the presence of human hosts in the vicinity of the AS at night. This finding highlights the persistent risk, albeit low, of JEV transmission in areas where Avian reservoir hosts are present. Most JEV infections would be asymptomatic in humans.^2^ Thus, it may be worthwhile to conduct seroprevalence studies in those residing close to such areas to estimate the true infection rate.

Interestingly, JEV was not detected after November 2011. There was a sharp decline in the total number of *Culex* spp. mosquitoes at all four sentinel sites after the months of November and December of 2011 (Figure 2). Trapping in February 2012 at the AS saw a reduction in both the total number of mosquitoes and *Cx. tritaeniorhynchus* by more than 50%. In Singapore, the number of mosquitoes in rural areas seems to be highly dependent on rainfall and the peaks coincide with the monsoon season at the end of the year. Therefore, the reduction in the number of mosquitoes at all four sentinel sites is likely to be seasonal. Enhanced vector control after notification of JEV detection at the AS may have also aided in the cessation of the virus transmission.

No WNV was detected in this study, corroborating with the absence of human cases in Singapore. Nevertheless, the importance of an effective surveillance system to monitor WNV geographical expansion cannot be ignored particularly after its point introduction into New York City and its subsequent southward spread in the Western hemisphere.^4^

From the NS5 phylogeny, SG/EHI-CV746 forms a novel species of CxFV that appears to be related most closely to the Quang Binh virus isolated in Vietnam in 2002 and CxFV-LSFlaviV-A20-09 virus isolated in China. In recent years, mosquito flaviviruses have been isolated from both *Aedes* and *Culex* mosquitoes in places such as Japan, China, Indonesia, United States, Mexico, Guatemala, and Trinidad.^43,44^ These viruses have not been attributed to any human diseases but are of great interest to virologists for studies on evolution as well as transmission and host specificity.^45^ They have also been shown to have the ability to enhance or suppress the replication of medically important flaviviruses.^46,47^ This could be of interest in future studies, given successful virus isolation.

Despite the absence of reported human JEV infection, JEV was detected in *Cx. tritaeniorhynchus*. Together with the presence of JEV antibodies in wildlife such as wild boar^22,26^ and birds,^22,26^ these new data strongly suggest the circulation of JEV among wildlife in Singapore. A One Health approach involving surveillance, the collaboration between public health and wildlife managers, and control of mosquito population remain the key measures in risk mitigation of JEV transmission in the enzootic cycle between birds and mosquitoes.
